# Lipid Transfer Proteins As Components of the Plant Innate Immune System: Structure, Functions, and Applications

**Published:** 2016

**Authors:** E. I. Finkina, D. N. Melnikova, I. V. Bogdanov, T. V. Ovchinnikova

**Affiliations:** Shemyakin and Ovchinnikov Institute of Bioorganic Chemistry, Miklukho-Maklaya Str. 16/10, 117997 , Moscow, Russia

**Keywords:** allergens, antimicrobial activity, cross reactivity, plant lipid transfer proteins, lipid binding and transfer, plant defense

## Abstract

Among a variety of molecular factors of the plant innate immune system, small proteins that transfer lipids and exhibit a broad spectrum of biological activities are of particular interest. These are lipid transfer proteins (LTPs). LTPs are interesting to researchers for three main features. The first feature is the ability of plant LTPs to bind and transfer lipids, whereby these proteins got their name and were combined into one class. The second feature is that LTPs are defense proteins that are components of plant innate immunity. The third feature is that LTPs constitute one of the most clinically important classes of plant allergens. In this review, we summarize the available data on the plant LTP structure, biological properties, diversity of functions, mechanisms of action, and practical applications, emphasizing their role in plant physiology and their significance in human life.

## INTRODUCTION

Lipids and their derivatives are involved in a variety of processes, including membrane biogenesis, cell differentiation, intercellular and intracellular signaling, and formation of water-repellent and thermal insulation covers protecting plants from adverse environmental factors; they also function as a storage and source of energy. The proteins involved in the intra- and extracellular transport of lipids play an important role in the lipid metabolism of pro- and eukaryotic cells. In plants, several classes of proteins capable of binding and transferring lipids and their derivatives have been identified: acyl-CoA-binding proteins; glycolipid-transfer proteins; sterol carrier proteins; homologues of the major pollen allergen of birch (*Betula verrucosa*), which is listed in the IUIS allergen database under the name Bet v 1; fatty acid binding proteins; puroindolines; and lipid transfer proteins.

Comparison of the amino acid sequences of the proteins of the listed classes demonstrated no significant structural homology among them. These proteins have an intra- or extracellular localization, relatively low molecular weight (7–30 kDa), a high isoelectric point (pI ~ 9–11), and a compact structure stabilized by disulfide bonds. A common feature of the spatial structure of lipid transfer proteins is a hydrophobic cavity accommodating a ligand-binding site. These proteins reversibly bind lipids and deliver them to their destination. Proteins of some classes have highly specific ligands, while other proteins bind and transfer a wide range of lipids.

LTPs belong to the most functionally important classes of plant proteins that bind and transfer lipids. These proteins were discovered in 1970 and were originally named phospholipid exchange proteins [1], but later they were renamed phospholipid transfer proteins [2]. Further studies showed that not only phospholipids, but other hydrophobic molecules as well may be ligands of such proteins, and, therefore, LTPs were given their present name – non-specific lipid transfer proteins [3].

## STRUCTURAL CHARACTERIZATION OF PLANT LTPS

**Fig. 1:**
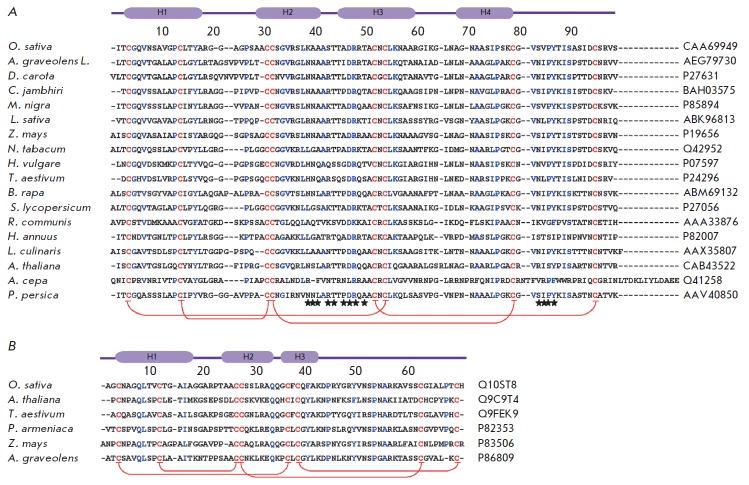
Comparison of the amino acid sequences of LTP1s (A) and LTP2s (B). Conserved cysteine residues are shown in red; amino acid residues typical of most subclass representatives are shown in blue. Disulfide bond arrangement is denoted by brackets. The localization of 4α-helices in the rice LTP1 (PDB ID: 1RZL) [5] and LTP2 (PDB ID: 1L6H) structures [10] is shown on the top of the panels. The asterisks denote the amino acid residues involved in the conformational epitopes of Pru p 3 (GenBank:AAV40850) [98].

On the basis of structural organization features, plant LTPs are divided into two subclasses: LTP1s with a molecular weight of 9–10 kDa and LTP2s with a molecular weight of about 7 kDa (*Table*). Amino acid sequence homology among representatives of the two subclasses is less than 30% (*Fig. 1*). All LTPs are basic proteins (pI ~9–10). The vast majority of LTPs contain eight conserved cysteine residues (..C^I^...C^II^...C^III^C^IV^... C^V^XC^VI^...C^VII^...C^VIII^..) forming four disulfide bonds that stabilize their structure and, thereby, underlie the resistance of LTPs to high temperatures and proteolytic enzymes. Some proteins from this class retain their native conformation and biological activity even after incubation at a temperature of about 100°C [4]. The LTP spatial structure is mainly composed of α-helical regions. Hydrophobic amino acid residues in LTPs are buried inside a molecule and are not in contact with each other, forming an internal protein cavity comprising a potential binding site for hydrophobic and amphiphilic molecules, such as lipids.

**Table:** Comparative characterization of two plant LTP subclasses

Characteristic	LTP1	LTP2
MW, kDa	9–10	6-7
Number of amino acids (a.a.)	90–95	65-70
Conserveda. a.	C, G, P, R, Y(F)	C, Q, P, Y(F)
–C^V^XC^VI^– motif	X – a hydrophilic amino acid residue (usually N) exposed on the protein surface	X – a hydrophobic amino acid residue (usually F) buried inside the protein molecule
Disulfide bond arrangement	C^I^–C^VI^, C^II^–C^III^, C^IV^–C^VII^, C^V^–C^VIII^	C^I^–C^V^, CII–C^III^, C^IV^–C^VII^, C^VI^–C^VIII^
Spatial structure	α-helices, a 3_10_-helix fragment, and an unstructured C-terminal loop	3 α-helices and a region containing a single helix-turn-helix
Hydrophobic cavity	A tunnel with large and small entrances that is formed by H1, H2, and H3 helices arranged parallel to each other	A triangular hollow box; H1 and H2 helices are arranged parallel to each other; the H3 helix forms an angle of 90° with H2
Sterol-binding ability	No	Yes
Amino acid residues interacting with a ligand	Arg44 and Tyr79 (numeration for rice LTP1)	Phe36, Tyr45, and Tyr48 (numeration for rice LTP2)
Signal peptide, a.a.	21–27	27–35
Localization	Cutin-coated organs (leaves, stems, flowers)	Suberin-coated organs (subterraneous organs)
Potential function	Cutin biosynthesis	Suberin biosynthesis
Activation of immune response	Elicitors in a complex with jasmonic acid	Elicitors in a complex with sterol
Allergens listed in IUIS	LTP1s of 42 plants (excluding iso-allergens and variants)	Tomato Sola l 6, celery Api g 6, peanut Ara h 16

**Fig. 2:**
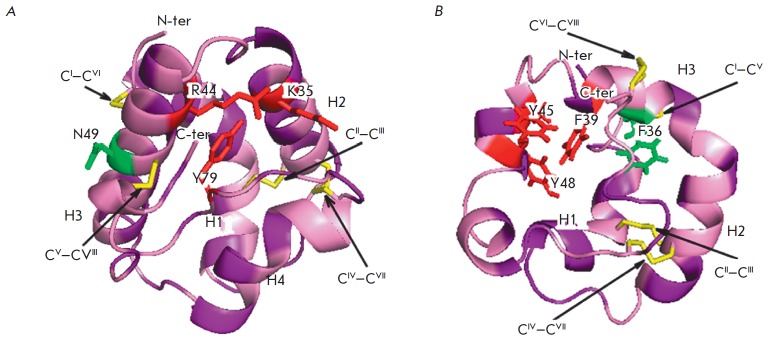
The spatial structures of (A) LTP1 (PDB ID: 1RZL) and (B) LTB2 (PDB ID: 1L6H) from rice in a ribbon representation. Identification numbers of α-helices (H1–H4) are specified. Hydrophobic amino acid residues are shown in purple; residues interacting with lipid ligands are shown in red [5, 10]; disulfide bonds are marked in yellow; the central residue in the –C^VX^C^VI^– fragment, directed either outward or inward the protein molecule, is shown in green.

LTP1s consist of 90–95 amino acid residues and have disulfide bonds formed in the following order: C^I^–C^VI^, C^II^–C^III^, C^IV^–C^VII^, and C^V^–C^VIII^ (*Fig. 1A*, *Fig. 2A*). The fragment –C^V^XC^VI^– in the LTP1 structure contains a hydrophilic amino acid (usually asparagine) whose side chain is exposed on the surface of a molecule. The spatial structure of these proteins consists of four α-helices, a 310-helix fragment, and an extended unstructured C-terminal region (*Fig. 2A*) [5, 6]. In the structure of some LTP1s, e.g., proteins isolated from maize *(Zea mays*) and tobacco (*Nicotiana tabacum*), the H1 and H4 helices are interrupted by proline residues into two fragments (H1a/H1b and H4a/H4b, respectively). The LTP1 hydrophobic cavity is shaped as an elongated tunnel formed by H1, H2, and H3 helices arranged parallel to each other. The hydrophobic nature of the tunnel surface is determined by the side chains of amino acid residues, including Ile, Val, Leu, and Ala; however, hydrophilic amino acid residues (Arg, Lys, Ser) are also involved in the cavity formation [7]. The tunnel in LTP1s has two entrances that differ in size. In most LTP1s, a basic residue – Arg44 – (position numbering relative to LTP1 of rice (*Oryza sativa*)) is located near the larger entrance and is involved in the interaction with polar lipid heads [8]. In rice LTP1, this interaction involves another basic residue, Lys35. In addition to cysteine residues, most LTP1s contain conserved glycine and proline residues that enable interhelical turns; two tyrosine residues, one of which is located in the N-terminal region, outside of the α-helix, and a second located in the C-terminal region, near the larger entrance to the hydrophobic tunnel and involved in the interaction with hydrophobic ligands [7, 9].

LTP2s consisting of 65–70 amino acid residues have been less studied than LTP1s. The –C^VX^C^VI^– fragment of LTP2s usually contains phenylalanine as the central residue, which points inward the molecule. LTP2s have a different organization of disulfide bonds: C^I^–C^V^, C^II^–C^III^, C^IV^–C^VII^, C^VI^–C^VIII^ (*Fig. 1B*, *Fig. 2B* spatial structure of proteins of this subclass includes three α-helices and a region containing single helical coils (*Fig. 2B*). In the LTP2 structure, the H1 and H2 helices are arranged parallel to each other and the H3 helix forms an angle of 90° with respect to H2. The shape of the LTP2 hydrophobic cavity resembles a triangular hollow box, with side chains of the Ala, Ile, Leu, Phe, and Val residues situated within. The volume of the triangular LTP2 box is smaller than that of the LTP1 hydrophobic cavity, but pronounced flexibility of the former allows proteins of this subclass to bind large ligands with a rigid structure, such as sterols [10-12]. Side chains of Phe39, Tyr45, and Tyr48 (numbering relative to rice LTP2) are rotated inside the cavity and in contact with a lipid ligand [13]. In addition to cysteine residues, the LTP2 structure comprises conserved Gln, Tyr, and Pro residues.

The hydrophobic cavity volume in both LTP subclasses can vary considerably. For example, the hydrophobic cavity volume of rice LTP1 is 249 A3, but the cavity volume increases to 1,354 A3 when the protein binds palmitic acid. This flexibility of LTP molecules may be the cause of their low specificity to a lipid ligand.

## LIPID BINDING AND TRANSFER

The presence of a hydrophobic cavity in the structure of LTP molecules enables these proteins to bind and transfer a variety of ligands. The LTP-ligand complex formation *in vitro *depends on the hydrophobic cavity size, the amino acid residues constituting the cavity, the spatial structure of the ligand, as well as experimental conditions (pH, buffer composition, temperature). LTPs isolated from various plant sources have been shown to be capable of binding lipids. However, it should be noted that there are exceptions to this rule. For example, a protein from onion (*Allium cepa*) seeds, termed Ace-AMP1, has a pronounced homology with plant LTPs but does not interact with lipids, perhaps because of the absence of a one-whole cavity in the protein molecule [14].

Various LTPs bind a wide range of ligands, including fatty acids (FAs) with a C_10_–C_18_ chain length, acyl derivatives of coenzyme A (CoA), phospho- and galactolipids, prostaglandin B2, sterols, molecules of organic solvents, and some drugs [15, 16]. Although LTPs lack marked specificity to ligands, these proteins form the most stable complexes with FAs containing from 16 to 18 carbon atoms. LTPs do not form stable complexes with molecules with a chain length longer than C_20_ due to the spatial constraints imposed by the hydrophobic cavity size [17]. Furthermore, the complex stability has been shown to be affected by the number of double bonds in FA molecules and their configuration. LTPs form the most stable complexes with various unsaturated FAs with one or two double bonds in the *cis*configuration [18], two of which, the linoleic and oleic acids, are precursors of cutin and suberin monomers.

Unlike LTP2s, LTP1s do not bind sterols. The ligand orientation in the LTP1 hydrophobic cavity was found to be different, depending on the spatial arrangement of ligand and LTP1 molecules. For example, in complexes of maize LTP1 with 1-palmitoyl lysophosphatidylcholine [9] and wheat (*Triticuma estivum*) LTP1 with dimyristoyl phosphatidylglycerol [18], ligands in the protein cavity occur in the “forward” orientation; i.e., polar lipid heads are located near the larger entrance to the hydrophobic cavity. At the same time, the ligand in the complex of barley (*Hordeum vulgare*) LTP1 with palmitoyl CoA occurs in “reverse” orientation, its aliphatic chains are strongly bent, and the polar head points towards the smaller entrance to the cavity [19].

Plant LTP1s can bind one or two lysophospholipid molecules [20]. LTPs of this subclass are supposed to interact with ligands according to a cooperative binding model. If two ligand molecules occur in the hydrophobic cavity, their orientation and binding affinity for the protein are not identical. For example, two lyso-myristoyl phosphatidylcholine molecules bound to wheat LTP1 have a “head-to-tail” orientation in the hydrophobic cavity [19, 21]. It is suggested that the second binding site of LTP is activated only when the first site is already occupied by a ligand.

The calcium-calmodulin system was shown to be involved in the regulation of lipid binding to plant LTPs. Plant LTPs bind calmodulin regardless of the presence of calcium ions. In maize Zm-LTP and onion Ace-AMP1, a potential site for binding of calmodulin is situated in the middle portion of the LTP polypeptide chain (residues 46–60) and has a structure similar to that of the basic amphiphilic α-helix (BAA) domain of calmodulin-binding proteins [22]. A distinctive feature of the BAA-like domain in plant LTPs is the absence of Trp that plays a crucial role in calcium-dependent binding of calmodulin. Maize Zm-LTP affinity for binding lipids is reduced in the presence of calmodulin. This is explained by the fact that the calmodulin binding site of the protein contains the Arg46 residue involved in the binding of lipids. At the same time, the calmodulin binding site in the bok choy (*Brassica rapa subsp. chinensis*) protein, termed BP-10, and arabidopsis (*Arabidopsis thaliana*) LTP1 is located in the C-terminal region (amino acid residues 69–81) and has no structural similarity with any of the known calmodulin binding sites [23]. The BP-10-calmodulin complex formation increases the efficiency of lipid binding. The cause of this effect is believed to be the residue Tyr81 located in the calmodulin binding site of the LTP protein and playing an important role in the interaction with a lipid ligand.

Plant LTPs not only bind lipids, but also transfer them between membranes in experiments *in vitro*. They transfer phospholipids, such as phosphatidylcholines (PCs), phosphatidylinositols (PIs), phosphatidylglycerols (PGs), their derivatives, as well as acyl-CoA [24-26]. Wheat LTPs were used to demonstrate that the lipid transfer activity of LTP2s is several times higher than that of LTP1s [27].

The lipid transfer mechanism involving LTPs remains unclear. Plant LTPs, like mammalian phosphatidylcholine- specific LTPs, are supposed to transfer lipids by the shuttle mechanism. A LTP-phospholipid complex interacts with the membrane, which results in phospholipid exchange between the complex and membrane [3].

To date, there is no direct evidence of involvement of plant LTPs in the binding and transfer of lipids *in vivo*. The only LTP-ligand complex found in plant cells is a covalent adduct of barley LTP1 and oxylipin that is formed by reacting the carboxyl group of Asp7 with the allene oxide in a 9(*S*),10-epoxy-10,12(*Z*)-octadecadienoic acid molecule [28, 29]. The reaction yields α-ketol-9-hydroxy-10-oxo-12(*Z*)-octadecenoic acid. It should be noted that the formation of this covalent complex, known as LTP1b, increases the hydrophobic cavity flexibility and the protein ability to transfer lipids.

Some LTPs are not only able to bind and transfer lipids but also to induce permeabilization of model membranes. For example, the sunflower (*Helianthus annuus*) protein termed Ha-AP10 damages liposomes consisting of PCs and PGs [30]. It is interesting to note the lack of a correlation between the lipid binding and lipid transfer activity and the LTP ability to damage membranes. For example, barley LTP binds a wide range of lipids but has little effect on the properties of model membranes [31]. Onion Ace-AMP1 does not bind lipids but induces permeabilization of bilayer vesicles consisting of anionic lipids [14].

## BIOSYNTHESIS AND LOCALIZATION

The LTP class belongs to a large family of pathogenesis- related proteins (PRPs). Induction of the synthesis of these proteins occurs upon exposure of a plant to abiotic and biotic stress factors and underlies one of the key defense mechanisms in plants. PRPs are present in all plant organs and accumulated in the vacuoles and apoplast, as well as in the primary and secondary cell walls. This localization is consistent with the defense function of PRPs that, along with antimicrobial peptides (AMPs), create a specific barrier to pathogen penetration [32].

The family of pathogenesis-related proteins includes, along with LTPs (PRP-14), proteins of 16 more classes: glucanases (PRP-2), chitinases (PRP-3, 4, 8), protease inhibitors (PRP-6), homologs of the major birch pollen allergen Bet v 1 (PRP-10), defensins (PRP-12), thionins (PRP-13), etc. [33]. Abiotic inducers of the PRP synthesis include UV radiation, osmotic shock, lack of moisture, low temperatures, and soil salinity. The PRP synthesis in an infected plant is induced by both primary and secondary elicitors: non-specific pathogen-associated molecular patterns (PAMPs) and damage-associated molecular patterns (DAMPs), as well as by specific effector proteins of pathogens. PRP synthesis inducers include phytohormones, such as ethylene, auxins, as well as abscisic, jasmonic, and salicylic acids. At certain stages of ontogeny, activation of synthesis and tissue-specific accumulation of PRPs also occur in the absence of stressors [34].

LTPs have been found in various plant organs: seeds, leaves, stems, roots, flowers, and fruits. Most often, LTPs occur in cuticle-covered epidermal cells but are also found in embryonic and vessel tissues. LTPs are synthesized in plant cells as preproteins containing a hydrophobic signal sequence (21–27 or 27–35 amino acid residues in LTP1s or LTP2s, respectively) and are secretory proteins with a predominantly extracellular localization [35, 36]. Some LTPs have an atypical intracellular localization. For example, LTP from castorbean (*Ricinus communis*) seeds was found in glyoxysomes [37]; LTP from cowpea (*Vigna unguiculata*) seeds was found in vacuoles [38]; Ca-LTP(1) from pepper (*Capsicum annuum*) seeds was found in vesicles [39]. Of particular interest is the question of how LTPs synthesized as preproteins without appropriate signal sequences occur in these cell organelles. Sunflower LTP, HaAP10, was found to be relocalized. In dry seeds, Ha-AP10 occurs in the apoplast; upon imbibition and germination of seeds, it relocalizes, possibly by endocytosis, to the intracellular organelles involved in lipid metabolism [40].

In some plants, LTPs termed GPI-anchored lipid transfer proteins (LTPGs) were found. These proteins are synthesized as precursors containing, in addition to the N-terminal signal peptide, the C-terminal signal sequence. This sequence ensures the post-translational attachment of the glycosylphosphatidylinositol anchor (GPI) to the protein, through which LTPGs can be localized on the outer side of the cell membrane or secreted to the apoplast after GPI-anchor cleavage [41]. Another group of unusual LTPs with extracellular localization is constituted by the xylogen from zinnia (*Zinnia elegans*) and xylogen-like proteins of other plants [42]. The gene structure of xylogen-like proteins, which belong to a large family of arabinogalactan proteins (AGPs), contains a signal peptide, the LTP domain, several AGP domains, and the GPI anchor attachment signal. During maturation, these proteins undergo a series of post-translational modifications, including removal of the N-terminal signal peptide, GPI anchor attachment, proline hydroxylation, and Oglycosylation [42].

Plant LTPs are encoded by multigene families and, in the plant genome, are usually represented by a set of genes encoding different isoforms. Expression of genes of different LTP isoforms is characterized by pronounced tissue specificity and occurs at certain stages of ontogeny [36]. This may be related to the fact that different LTP isoforms have different functions [43]. Differential expression of genes of multiple LTP isoforms also occurs when a plant is exposed to a variety of abiotic and biotic environmental factors and may be considered as one of the defensive strategy elements under stress conditions [44]. Differential expression of isoform genes was shown for LTPs from sesame (*Sesam umindicum*) [45], arabidopsis [43, 46], pepper [47], castorbean [37], grape (*Vitis vinifera*) [48], Kashgar tamarisk (*Tamarix hispida*) [49], and tomato (*Lycopersicon pennellii*) [50].

## BIOLOGICAL ACTIVITY

As mentioned, LTPs constitute one of the classes of defense PRPs, many of which have antimicrobial and enzymatic activities or are enzyme inhibitors. Various representatives of the LTP class exhibit antibacterial, antifungal, antiviral, and antiproliferative activities, and inhibit some enzymes [36].

**Antimicrobial activity**

Many LTPs have antimicrobial activity and inhibit the growth of pathogenic bacteria and fungi, such as* Clavibacter michiganensis*, *Pseudomonas solanacearum*,* P. syringae*, *Alternaria brassicola*, *Ascochyta pisi*,* Colletotrichum lindemuthianum*, *Fusarium solani*, *F. graminearum*, *F. culmorum*, *F. oxysporum*, *Botrytis cinerea*, *Sclerotinia sclerotiorum*, *Verticillium dahliae*, etc. LTPs from pepper and coffee (*Coffe acanephora*) are also active against human pathogenic fungal strains from the *Candida *genus [39, 51]. The antimicrobial activity of most plant LTPs is specific and exhibited against a particular spectrum of microorganisms. LTPs from onion [52], radish (*Raphanus sativus*) [52], and arabidopsis [53] have pronounced antimicrobial activity at micromolar concentrations. Most LTPs have a moderate or little effect on the growth of microorganisms; in some cases, this effect is absent. [54] The antimicrobial activity of plant LTPs decreases in high salt solutions and in the presence of calcium ions, which is a common feature of other classes of plant AMPs and PRPs [52]. Like plant defensins, LTPs are able to act in synergy with thionins [55] and have no toxic effects on plant cells and mammalian cells, including fibroblasts and red blood cells [30, 52].

Disruption of the disulfide bonds stabilizing the structure of plant LTPs leads to a loss of the ability of the proteins to inhibit the growth of microorganisms and bind lipids [56]. At the same time, the other amino acid residues that are necessary for exhibiting the antimicrobial activity remain unknown. The antimicrobial activity of rice LTP110 was shown to require the presence of the conserved residues Tyr17, Arg46, and Pro72 that play an important role in the stabilization of the protein structure in most LTP1s [57]. A study of wheat LTP isoforms demonstrated that difference in one amino acid residue only (Pro3Ser in TaLt10B6 and TaLt710H24 isoforms and Asn24Ser in TaLt10F9 and TaBs116G9 isoforms) significantly affects the antimicrobial activity of the proteins. It is assumed that the replacement of just one amino acid residue may result in a change in the LTP spatial structure and affect the positive charge distribution over the molecule surface [56].

To date, the antimicrobial activity of plant LTPs is found not to be related to their ability to interact with lipids. For example, eight wheat LTP isoforms were shown to have no correlation between the ability of the proteins to inhibit the growth of pathogenic microorganisms and to bind lipids [56]. Using onion Ace-AMP1 [52] and a mutant rice LTP isoform [57], it was also shown that this class of proteins may possess antimicrobial activity but not bind lipid molecules and vice versa.

Plant LTPs have not only fungistatic, but also fungicidal activity and, like other AMPs, are able to induce permeabilization of the model membranes [30] and cell membranes of pathogenic fungi [30, 56]. For example, LTPs from onion [14], sunflower [30], and, to a lesser extent, barley [31] are able to induce permeabilization of liposomes consisting of anionic phospholipids only or a mixture of anionic and neutral phospholipids, causing fluorescent dye leakage from liposomes. However, it should be noted that this effect is much weaker than that in other plant AMPs and observed only in lowionic- strength solutions.

The mechanism of antimicrobial action of representatives of the LTP class remains unclear. Nevertheless, the cell membrane is considered as a potential target for LTP antimicrobial action. Plant LTPs, like other cationic membrane-active AMPs, are supposed to bind to the cell membrane of the phytopathogen through electrostatic interactions and cause destabilization and permeabilization of the membrane. The weaker antimicrobial activity of LTP isoforms containing a smaller number of basic amino acids is explained by the attenuation of the electrostatic interaction with the cell membrane of the phytopathogen [56]. A potential cause of the selective toxicity of plant LTPs is believed to be the differences in the lipid composition of the cell membranes of bacteria, fungi, plants, and mammals.

**Antiviral and antiproliferative activities**

LTPs from Chinese daffodil (*Narcissus tazetta*) and cole seed (*Brassica campestris*) were shown to have antiviral activity and the ability to inhibit the proliferation of human tumor cells. In *in vitro *experiments, *N.tazetta* LTP, designated as NTP, significantly inhibited plaque formation of the respiratory syncytial virus (RSV), the cytopathic effect of the influenza A virus (H1N1), and the proliferation of the human acutepromyelocytic leukemia cell (HL-60). *B. campestris *LTP inhibit the activity of HIV-1 reverse transcriptase and the proliferation of hepatoma HepG2 and breast cancer MCF7 cells. To date, the mechanism of LTP anti-tumor activity has not been determined [58, 59].

**Inhibition of enzyme activity**

Some members of the LTP class, like protease inhibitors (PRP-6) and certain defensins (PRP-12) [60, 61], can inhibit the activity of proteolytic enzymes and α-amylases. For example, barley seed LTPs of both subclasses were found to inhibit cysteine endoproteases [62]. Also, LTP1 from the *Ginkgo biloba *seed inhibits cysteine (papain), aspartate (pepsin), and serine (trypsin) proteases [63]. LTP1 from seeds of coffee and pepper inhibit the activity of human α-amylase [39, 51]. LTPs capable of inhibiting the activity of their own and foreign enzymes are believed to be involved both in the development and germination of seeds and in the protection of plants against insects and herbivores.

## POTENTIAL LTP FUNCTIONS

**Fig. 3:**
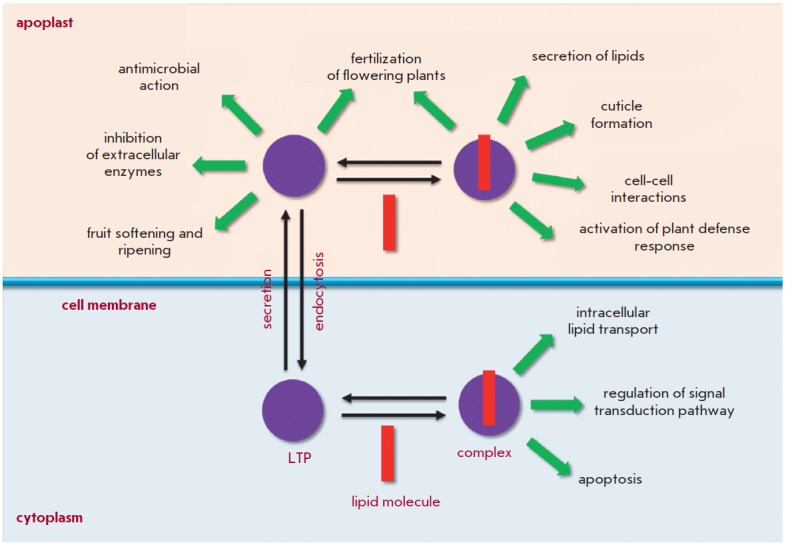
Potential LTP functions in plants

LTPs are known to play an important role in plants. Knockout of the genes encoding these proteins leads to disruption of the vegetative and reproductive development of plants and a decrease in their resistance to infections [43, 64, 65]. The results of a study of the inhibition of LTP gene expression support a number of assumptions about the possible involvement of proteins from this class in the adaptation of plants to stress, lipid metabolism, embryogenesis, growth and reproduction of plants, symbiosis, and other processes. Many of these functions are believed to be associated with the LTP ability to bind and transfer lipid molecules (*Fig. 3*).

**Involvement in lipid metabolism**

Because plant LTPs are capable of binding and transferring lipids, these proteins are believed to be involved in a variety of processes that are accompanied by changes in lipid composition. Extracellular LTPs are supposed to participate in the formation of a protective cuticle layer whose monomeric components are formed in epidermal cells and delivered to the biosynthesis site. Activation of biosynthesis of the cuticle, which plays an important role in maintaining the water balance and protection of plants from penetration by pathogens, occurs under the action of a variety of stress factors and is one of the defense mechanisms in plants. There is no direct evidence of LTP involvement in this process. However, plant LTPs were shown to occur at high concentrations in epidermal tissues and of being capable of binding the fatty acids required for the synthesis of cutin and suberin. Furthermore, induction of LTP synthesis is accompanied by thickening of the cuticle layer [66] and knockout of LTP genes leads to changes in the lipid composition and density of the cuticle layer [67]. Two potential mechanisms of cuticle component delivery with involvement of LTPs were suggested. According to the first of them, LTPs enter the cell by receptor-mediated endocytosis and are loaded by the fusion of vesicles containing LTP and cutin monomers. The second mechanism suggests shuttling of LTPs between the cell membrane and the cell wall of plants and the existence of a carrier molecule acting on the inner side of the cell membrane [68]. An interesting fact is that LTP1s are present in organs covered by a cutin layer (leaves, stems, flowers), while LTP2s occur in suberin- covered subterranean organs. This argues for a differential involvement of proteins of the first and second subclasses in the cutin and suberin layer formation [35]. LTPGs having a GPI anchor were demonstrated to be possibly involved in the biosynthesis and accumulation of suberin [41].

LTPs found in various intracellular organelles are presumably involved in the mobilization of lipids through their transfer, e.g., during seed germination. For example, castorbean LTP found in glyoxysomes binds both free FAs and acyl-CoA. This protein also increases the activity of acyl-CoA oxidase involved in the β-oxidation of FAs [37]. Sunflower LTP Ha-AP10 entering the cell during seed germination is supposed to transfer FAs, liberated by cleavage of triacylglycerols, to glyoxysomes for further β-oxidation [40].

Induction of the expression of genes encoding carrot (*Daucus carota*) LTP was demonstrated to occur at the early stages of embryogenesis when degradation of some lipids and biosynthesis of others, as well as the protective lipid layer formation around the embryo, takes place [69]. The role of this protein in embryogenesis is presumably to participate in these processes via the transfer of relevant lipid molecules.

**Involvement in fertilization of flowering plants**

Plant LTPs are believed to play an important role in the reproduction of flowering plants. For example, lily (*Lilium longiflorum*) LTP1 is a component necessary for pollen adhesion and formation and growth of the pollen tube [70]. LTP1 is supposed to be capable of acting directly as an adhesive component or as a carrier of the hydrophobic adhesive component. Also, one of the isoforms of a lipid transfer protein from arabidopsis, LTP5, was shown to be involved in the growth of the pollen tube and seed formation [64].

The role of rice LTP OsC6 in postmeiotic pollen development has been determined. This protein was found to be present in anther tissue and to be capable of binding FAs. OsC6 is supposed to be involved in the formation of lipid orbicles and pollen exine through transfer of essential lipids from tapetum cells to microspores [65].

**Involvement in protection and adaptation of plants under stress conditions**

The belief that LTPs are involved in the protection and adaptation of plants to stress is mainly based on the fact of a stress-induced synthesis of these proteins. For example, the synthesis of LTPs, as well as that of other PRPs, is induced by wounding, moisture deficit, low temperatures, soil salinity, infections, and chemical agents [43, 45, 47, 50, 71, 72]. Induction of the expression of LTP genes under stress conditions may be associated with the presence of regulatory elements, which are also typical of other PRPs, in the promoter region of LTP genes. The regulation of LTP gene expression involves phytohormones, such as abscisic and salicylic acids, ethylene, and methyl jasmonate [36].

One of the possible causes behind the induction of LTP gene expression under stress conditions is believed to be the involvement of these proteins in the biosynthesis of the cuticle layer [50]. The protective function of LTPs in plants is related to their antimicrobial activity, cryoprotective action, and their ability to inhibit exogenous enzymes, as well as to their possible involvement in the secretion of other components of the plant immune system.

The glandular hairs (trichomes) of plants produce essential oils that are involved in metabolism, protect plants against pests and overheating, have a woundhealing effect, and attract insects. Tobacco (*N. tabacum*) NtLTP1 was found to be specifically expressed in long glandular trichomes and to be involved in the secretion, from trichome heads, of essential oil components (diterpenes, aliphatic hydrocarbons, and aromatic acids) that are plant protective factors [73]. LTP gene transcripts were also found in the glandular hairs of other plants, such as pepper mint (*Mentha piperita*), alfalfa (*Medicago sativa*), sweet wormwood (*Artemisia annua*), hop (*Humulus lupulus*), Greek sage (*Salvia fruticosa*), and tomato [73].

The resistance of plants to cold is known to be associated with stabilization of cell membranes and prevention of a protein solubility reduction at lower temperatures. WAX9 proteins that have a high degree of amino acid sequence homology with LTPs were identified in the leaves of a cold-acclimated cabbage (*Brassica oleracea*). These proteins cannot bind lipids, but, like β-1,3- glucanases, osmotins, and lectins, they are able to stabilize thylakoid membranes in cold conditions [72]. The mechanism of cryoprotective action of these proteins is supposed to be associated with a decrease in the fluidity of membrane lipids upon interaction between LTPs and the thylakoid membrane [74].

**Involvement in activation and regulation of signaling cascades**

LTPs are supposed to be involved in the activation and regulation of various signaling pathways in plants through the formation of complexes with various lipid molecules. Oxylipins are one of the classes of signal mediators in plants. Oxylipins are produced from unsaturated FAs under the action of reactive oxygen species (ROS) or enzymes and are involved in the regulation of the growth and development of plants, as well as in triggering defense responses to stress conditions. In addition, oxylipins regulate the processes of neutralization of the toxic components formed during stress. As mentioned, barley LTP1, during seed germination, forms covalent complexes with oxylipin of 9(*S*),10-epoxy-10,12(*Z*)-octadecadienoic acid containing an unstable allene oxide resulting from the sequential action of lipoxygenase and allene oxide synthase [28, 29]. This interaction may indicate a joint involvement of LTPs and oxylipins in the regulation of the signaling pathways that trigger the mechanism preventing damage to plant cells under stress conditions [29].

**Fig. 4:**
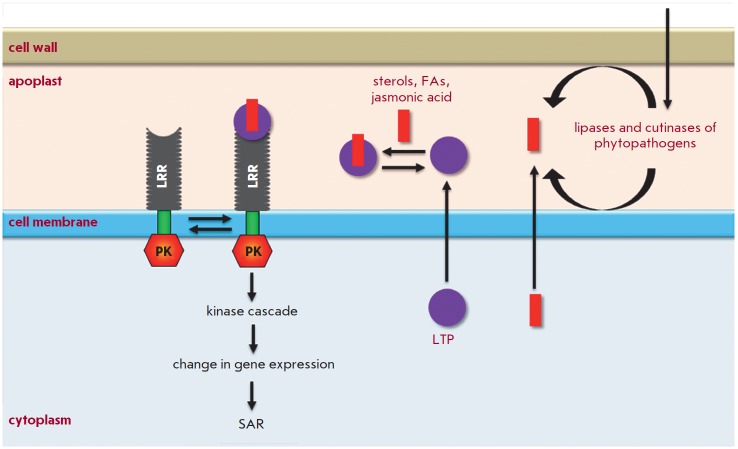
A potential mechanism of LTP involvement in plant immune response. LTPs are secreted to the apoplast and bind to lipid molecules that are either secreted by the plant (e.g., jasmonic acid) or form under the action of enzymes secreted by phytopathogenic microorganisms. Lipid-bound LTPs interact with receptors, such as receptor-like serine/ threonine protein kinases, located on the cell membrane that contain the extracellular leucine-rich repeat domain (LRR), transmembrane, and cytoplasmic protein kinase (PK) domains. This interaction may cause signal transduction mediated by versatile second messengers and a cascade of mitogen-activated protein kinases; activation of several transcription factors; induction of the synthesis of protective factors, including AMPs and PRPs (possibly, LTP isoforms with a pronounced antimicrobial activity); and, finally, SAR.

LTPs bound to lipid molecules act as endogenous elicitors interacting with specific receptors on the cell membrane of plant cells and providing for the development of an immune response to infection (*Fig. 4*). For example, rice and tobacco LTPs were shown to be capable of interacting with elicitin receptors [21, 75, 76]. Elicitins are well-studied plant PAMPs that have a molecular weight of about 10 kDa and are produced by phytopathogenic oomycetes (*Phytophthora *and *Pythium*) parasitizing on higher plants. These proteins, due to a hydrophobic cavity in their structure, can bind sterols and provide phytopathogenic microorganisms with essential plant-derived lipids. All elicitins have a α-helical structure stabilized by three disulfide bonds; sterol-associated elicitins are recognized by the plant by means of receptor-like kinases located on the cell membrane. The recognition entails activation of plant defense mechanisms, such as the production of phytoalexins and ROS, as well as the development of a hypersensitive response (HR) and systemic-acquired resistance (SAR) [77, 78]. The amino acid sequences of LTPs and elicitins have a low degree of homology, whereas the spatial structures of the proteins have a pronounced similarity [79]. Lipid bound plant LTPs act as agonists of elicitins and DAMP, bind to elicitin receptors, and trigger an immune response. An interesting fact indicating the possibility of different pathways for the activation of a plant defense response involving representatives of the two LTP subclasses is the difference in the structure of a hydrophobic ligand. Sterols act as this ligand for LTP2s [75], while jasmonic acid is the ligand for LTP1s that have a less flexible hydrophobic cavity [21, 76].

An unusual LTP2 representative from arabidopsis – termed DIR1 – that has an isoelectric point in the acidic pH range plays the key role in SAR development [80]. During plant infection, the protein is supposed to bind to the lipid molecules (oxylipins, fatty acids, or monoacyl phospholipids) produced by lipases secreted by the pathogen. Then, the formed complex interacts with a hypothetical receptor, triggering a signaling cascade that leads to SAR development [81].

Zinnia xylogen containing the GPI anchor and binding plant sterols was found to promote the differentiation of uncommitted cells into tracheary elements and, probably, to be involved in cell-cell interactions and signal transduction. It is thought that xylogen-like proteins of other plants, whose LTP domains are highly similar to those of LTP2s, may also be involved in cellcell interactions and signal transduction, functioning in a complex with a lipid molecule [42].

**Involvement in apoptosis**

Possible LTP involvement in apoptosis was assumed based on the similarity between maize LTP and the mammalian pro-apoptotic protein Bid that also has an internal cavity and binds and transfers lipids [82]. Bid occurs in the cytosol and, in the presence of lysophospholipids generated during programmed cell death, affects mitochondria, causing the release of apoptogenic factors, including cytochrome *c*. In the presence of lysophospholipids, maize LTP also causes the release of cytochrome *c *from mitochondria. A possible mechanism of the destabilizing action of both proteins includes transfer of lysophospholipids to the outer mitochondrial membrane. The lysophospholipids modify the membrane properties, thereby facilitating the action of other pro-apoptotic proteins [83].

**Involvement in symbiosis**

Symbiotic rhizobacteria are known to be able to stimulate the growth of plants and protect them from soil phytopathogens, causing the development of the socalled induced systemic resistance (ISR) that is phenotypically and functionally similar to SAR [84]. Alfalfa LTP MtN5 was shown to play an important role in the development of symbiotic relationships between a plant and nodule bacteria. Namely, the protein is involved in processes of bacteria penetration into root tissues and nodule formation [85]. The MtN5 function is supposed to maintain the balance between bacterial invasion and prevention of infection [86].

**Involvement in fruit ripening**

Tomato LTP was shown to be capable of forming complexes with polygalacturonase, which is the most significant pectin-degrading enzyme. Upon complex formation, tomato LTP enhances the hydrolytic activity of the enzyme and may be involved in the regulation of fruit softening and ripening [87].

## LTPs AS ALLERGENS

LTPs are antigens involved in the development of allergic reactions of varying severity to pollen, plant foods, and latex. The structure of these proteins, stabilized by disulfide bonds, is responsible for their high resistance to cleavage by digestive enzymes and enables the proteins to reach the human intestine in native immunogenic form and to cause sensitization [88]. The allergenic capacity of LTPs in various processed foods (juices, jams, beer, wine, etc) is explained by their highly stable structure that is practically unsusceptible to thermal denaturation, as well as chemical and enzymatic degradation [89]. It should be noted that the defined allergens are mostly members of the first subclass of plant LTPs. For example, the IUIS allergen database now contains only three LTP2s (tomato, peanut, and celery) and 42 LTP1s from various plants, apart from their isoforms. The high structural homology of LTP1s underpins the development of cross-allergic reactions.

LTP1s, which are widely distributed in the plant kingdom, are the main allergens isolated from fruits and grains (peach (*Prunus persica*) Pru p 3, cherry (*P. avium*) Pru av 3, apple (*Malus domestica*) Mal d 3, plum (*P. domestica*) Pru d 3, orange (*Citrus sinensis*) Cit s 3, grape Vit v 1, strawberry (*Fragaria ananassa*) Fra a 3), nuts (hazelnut (*Corylus avellana*) Cor a 8, walnut (*Juglans regia*) Jug r 3, chestnut (*Castanea sativa*) Cas s 8), vegetables (asparagus (*Asparagus officinalis*) Aspa o 1, lettuce (*Lactuca sativa*) Lec s 1, cabbage Bra o 3, tomato (*Lycopersicone sculentum*) Lyc e 3, celery (*Apium graveolens*) Api g 2), cereals (maize Zea m 14, rice Ory s 14, wheat Tri a 14, barley Hor v 14), and legumes (peanut (*Arachis hypogaea*) Ara h 9, lentil Len c 3, bean Pha v 3) [90-92]. It is important to note that LTPs are accumulated mainly in the skin of fruits, not in their pulp [93], which may be the cause of anaphylactic reactions in humans upon dermal contact with fruits [94]. A significant contribution to primary sensitization is also made by pollen allergens of the LTP class: Jewish pellitory (*Parietaria judaica*) Par j 2, olive (*Olea europaea*) Ole e 7, plane tree (*Platan usacerifolia*) Pla a 3, mugwort(*Artemisia vulgaris*) Art v 3, etc. [95]. Interestingly, LTPs from fruits of *Rosaceae *family plants were also found in the pollen of these trees [96]. Peach Pru p 3 is believed to be the main LTP allergen that plays the major role in sensitization and is recognized by immunoglobulin E (IgE) in most individuals with allergies [97, 98].

In recent years, numerous studies have been conducted to elucidate the causes of high plant LTP1 allergenicity and development of cross-induced allergic reactions. For example, IgE-binding B-cell epitopes of Pru p 3 were identified. These are positively charged moieties on the protein surface that are associated with the amino acid residues 11–25, 31–45, and 71–80 (*Fig. 1A*) [99]. The identified antigenic determinants are highly homologous among various allergenic LTP1s. The key role in the interaction between Pru p 3 and IgE was found to be played by the residues Arg39, Thr40, and Arg44 that are typical of most allergenic LTP1s [100]. A polypeptide chain fragment comprising the amino acid residues 61–80 acts as a T-cell epitope of Pru p 3 [101]. Also, the development of a T-cell response to Pru p 3 was shown to be accompanied by increased expression of integrin α4β7 that provides lymphocyte migration to the intestinal wall where the primary lymphocyte activation occurs [102].

## EVOLUTION OF GENES

LTP genes are ubiquitous in higher plant genomes: from the most primitive bryophytes to tracheophytes, including ferns, lycopsids, angiosperms, and gymnosperms, but they are not found in lower plants, such as algae. In this regard, it is assumed that the LTPs involved in the protection of plants against various environmental stress factors could have developed during the emergence of terrestrial plants, i.e. about 400 million years ago [103].

As mentioned above, LTPs of one plant are usually encoded by tens of related genes forming a multigene family. The emergence of multiple LTP isoforms performing different functions in plants during evolution is believed to be associated with a number of successive duplications of an ancestral gene and subsequent mutations [104]. During evolution, most angiosperms are known to undergo one or more duplications of the whole genome. A phylogenetic analysis of multiple LTP isoforms of rice, wheat, and arabidopsis indicates that duplication of genes and chromosome fragments continues at the present time [105]. During evolution, mutations in duplicated LTP genes could lead to gene pseudogenization, subfunctionalization with preservation of some functions of the ancestral gene, or neofunctionalization, i.e. acquisition of totally new functions by the gene [106]. The last two possibilities might lead to the appearance of new LTP isoforms with a different spectrum and degree of biological activity, as well as LTP-like proteins that significantly differ from members of the LTP class in structure and perform other functions.

## PRACTICAL APPLICATIONS

**LTPs as drug carriers**

The LTP ability to bind and transfer lipids creates opportunities for possible use as ligand-binding proteins in developing drug and cosmetic agent delivery systems that protect from premature biodegradation or reduce side effects upon systemic application. The possibility of developing a LTP-based delivery system depends on a number of LTP properties: a) resistance to heat denaturation and protease action; b) the hydrophilic surface ensuring biocompatibility of a LTP-ligand complex and a reduced risk of side reactions; c) protection of a drug disposed within the LTP hydrophobic cavity from premature biodegradation; d) the small size of a LTP-ligand complex, ensuring its effective penetration into tissues; and e) increased affinity to and specificity for LTP-ligand complex formation, which may be achieved by modifying the protein amino acid sequence.

Several studies have demonstrated that plant LTPs form complexes not only with FAs and phospholipids, but also with other hydrophobic and amphiphilic ligands, including some drug substances. For example, wheat LTP1 forms complexes with prostaglandin B2 (PGB2). Upon interaction with LTP1, PGB2 was found to immerse completely into the hydrophobic cavity of the protein, becoming isolated from the environment [17]. Wheat LTP1 was shown to bind some components of the skin lipid layer (sphingosines, sphingomyelins, and cerebrosides), which are used in cosmetics. Thus, wheat LTP1 may be used in cosmetology as a skin lipids carrier. On the other hand, wheat LTP1 is able to bind drugs that are active against pathogens of leishmaniasis and HIV-1 and exhibit antineoplastic properties, but have serious side effects when administered systemically (e.g., edelfosine, ilmofosine, and their analogs). Using wheat LTP1 as a delivery vehicle may significantly reduce the toxicity of these drugs. Furthermore, wheat LTP1 is able to deliver antifungal agents, such as conazole BD56 and amphotericin B [16]. It should be noted that the protein binds all these substances with low affinity, which is a prerequisite for the transport and controlled release of the ligand.

Screening of maize LTP1 and rice LTP2 using the Comprehensive Medicinal Chemistry (CMC) database containing information about 7,300 biologically active compounds demonstrated that the proteins contain not one but two potential drug binding sites: one site occurs in the hydrophobic cavity, while the second site is situated on the hydrophilic surface of the protein molecule. In rice LTP2, the binding site for sterols, such as β-sitosterol or cholesterol, is located near the hydrophobic cavity; the binding site for triphenylmethane derivatives, such as diphenyl-4-pyridylmethane, occurs on the protein surface, near the C-terminal region [15].

**LTPs in the food industry**

Surfactant properties of plant LTPs enable their use in the food industry as emulsion and foam stabilizers. Beer brewing is one of the food industry sectors where these LTP properties are widely used. The formation and stability of foam are known to be important beer quality indicators. Numerous studies demonstrate that LTPs are the major protein components of barley beer and play the key role in the formation and stabilization of beer foam [35, 75]. The main beer components include a barley LTP1 protein that binds lipids and, thereby, reduces their negative impact on the formation and stability of foam. In the brewing process, LTP1 glycosylation and acylation occur, which increases amphiphilicity and the surfactant properties of the protein [75]. LTP1b, a LTP1-9(*S*),10-epoxy-10,12(*Z*)-octadecadienoic acid covalent complex, forms during fermentation, which was mentioned above [107]. LTP1 and LTP1b are resistant to high temperatures and retain their structure and ability to interact with lipids upon heating during beer pasteurization. It should be noted that LTP1, unlike LTP1b, has antifungal activity, inhibits growth of yeast, and, therefore, can adversely affect the fermentation process. Therefore, LTP1b formation and the equilibrium between free and lipid- bound forms of LTP1 in beer are important for brewing high-quality barley beer.

**Generation of viable transgenic plants**

Of high interest is the possibility of using LTPs for generating transgenic plants resistant to various abiotic and biotic stress factors. Transgenic plants carrying LTP genes possess enhanced resistance to phytopathogenic microorganisms [108], pests [73], high temperatures [109], soil salinity [108], drought [110], etc.

**LTPs in allergology**

Another promising application for natural and recombinant plant LTPs is the development of modern test systems for component-resolved allergy diagnostics and vaccines for preventive allergen-specific immunotherapy (ASIT).

The main methods of allergy diagnostics include skin-provocative tests and elimination diet, together with enzyme immunoassay or immunofluorescent analysis aimed at assessing the total and specific IgE and IgG antibodies levels. Classical allergy diagnostics uses crude allergen extracts yielding poorly reproducible, and sometimes even false, results due to the lack of a possibility to standardize them and fluctuations in the content of allergenic proteins and non-protein components. The current direction in allergy diagnostics development is based on the replacement of crude extracts by individual allergic components, which can be used to produce a molecular profile of the patient’s sensitivity and to study cross-reactivity [111]. Modern microarray-based test systems designed for component- resolved diagnostics use several natural and recombinant pollen (mugwort Art v 3, plane tree Pla a 3, pellitory Par j 2, and olive Ole e 7) and food (peach Pru p 3, hazel Cor a 8, walnut Jug r 3, peanut Ara h 9, and wheat Tri a 14) allergenic LTPs.

A modern method for reducing the reactivity of an organism is allergen-specific immunotherapy (ASIT), where the patient is administered gradually increased allergen doses [112]. However, classical ASIT uses crude extracts or allergoids that have a low efficacy and a high risk of systemic allergic reactions. The most safe and promising ASIT approach involves the design and development of vaccines on the basis of individual natural and recombinant allergens and their hypoallergenic analogs. These analogs should have low allergenicity but quite high immunogenicity to avoid adverse allergic reactions and reduce the hypersensitivity for a long time [113]. Hypoallergenic forms are developed mainly using methods of rational design and site-directed mutagenesis by replacing amino acid residues constituting B-cell epitopes. To date, several hypoallergenic analogs of major pollen and food allergens from different classes are undergoing clinical trials [114]. So far, hypoallergenic forms of some plant LTPs have been produced: e.g., pellitory Par j 2 [115] and peach Pru p 3 [116]. However, there are no vaccines on the basis of hypoallergenic forms of plant LTPs among the drugs under clinical trials.

## CONCLUSION

LTPs are widespread in the plant kingdom and present in almost all plant tissues and organs, have intraor extracellular localization, and play an important physiological role. LTPs encoded by a multigene family in plants are represented by a set of multiple isoforms differentially expressed in various tissues and organs under the influence of various stress environmental factors. In addition, various LTP-like proteins with very different structures and functional activities have been found in plants. The emergence of multiple isoforms of LTPs and LTP-like proteins during evolution is assumed to result from the need to expand the range of functions of these proteins.

The biological role of LTPs in plants is poorly understood. LTPs have been demonstrated to be involved in many processes, which might be largely associated with their ability to bind and transfer a variety of lipid molecules.

LTPs have been reliably ascertained to belong to molecular factors of the plant innate immune system. As components of the PRP family, LTPs belong to the plant defense system that enables them to adapt quickly and survive under stress conditions. The defense function of LTPs is associated with their antimicrobial activity and ability to inhibit foreign enzymes, involvement in the transfer of signaling mediators and protective and building lipids, as well as with their properties as endogenous elicitors whose complexes with lipids are recognized by specific receptors and trigger an immune response.

LTPs play an important role in human life. Their widespread occurrence and a similar spatial organization make these proteins one of the most important classes of cross-reactive plant allergens that are a frequent cause of allergic reactions of varying severity. Their surfactant and allergenic properties, as well as the LTP ability to bind and transfer hydrophobic ligands, make it possible to use these proteins in pharmacy for designing drug and cosmetic agent delivery systems; in allergology, for developing modern diagnostic test kits and vaccines for ASIT; in the food industry, for brewing high-quality beers; and in agriculture, for generating stress-resistant plants.

## Abbreviations

LTP: lipid transfer protein
FA: fatty acid
PC: phosphatidylcholine
PI: phosphatidylinositol;
PG: phosphatidylglycerol
PRP: pathogenesis-related protein
AMP: antimicrobial peptide
PAMP: pathogen associated molecular pattern
DAMP: damage associated molecular pattern
GPI anchor: glycosylphosphatidylinositol anchor
ROS: reactive oxygen species
SAR: systemic acquired resistance
HR: hypersensitive response
ASIT: allergen-specific immunotherapy
